# In a Safety Net Population HPV4 Vaccine Adherence Worsens as BMI Increases

**DOI:** 10.1371/journal.pone.0103172

**Published:** 2014-07-30

**Authors:** Diane M. Harper, Britney M. Else, Mitchell J. Bartley, Anne M. Arey, Angela L. Barnett, Beth E. Rosemergey, Christopher A. Paynter, Inge Verdenius, Sean M. Harper, George D. Harris, Jennifer A. Groner, Gerard J. Malnar, Jeffrey Wall, Aaron J. Bonham

**Affiliations:** 1 Department of Community and Family Medicine, University of Missouri-Kansas City School of Medicine, Kansas City, Missouri, United States of America; 2 Department of Obstetrics and Gynecology, University of Missouri-Kansas City School of Medicine, Kansas City, Missouri, United States of America; 3 Department of Biomedical and Health Informatics, University of Missouri-Kansas City School of Medicine, Kansas City, Missouri, United States of America; 4 Department of Obstetrics and Gynecology, Radboud University, Nijmegen, the Netherlands; 5 Hampshire College, Amherst, Massachusetts, United States of America; Centers for Disease Control and Prevention, United States of America

## Abstract

**Objectives:**

Obesity adversely inhibits antibody response to vaccination. Three doses of HPV4 may or may not provide adequate long term protection against HPV 16/18 in obese females. The aim of this study was to determine whether adherence to HPV4 vaccination in a safety net population was reduced with increasing body mass index (BMI).

**Methods:**

We designed a historical prospective study evaluating the number and dates of HPV4 dosing that occurred from July 1, 2006 through October 1, 2009 by the demographic characteristics of the 10–26 year old recipient females. The defined dosing intervals were adapted from the literature and obesity categories were defined by the WHO.

**Results:**

1240 females with BMI measurements received at least one dose of HPV4; 38% were obese (class I, II and III) and 25% were overweight. Females with normal BMI received on-time triplet dosing significantly more often than did the obese class II and III females (30% vs. 18%, p<0.001). Obese class II/III females have a significant 45% less chance of completing the on-time triplet HPV4 series than normal women (OR = 0.55, 95% CI: 0.37, 0.83). Pregnancy history has a significant influence on BMI and HPV4 dosing compliance in this safety net population where 71% had been gravid. Hispanic females were less likely to complete HPV4 dosing regardless of BMI (aOR = 0.39, 95% CI: 0.16, 0.95).

**Conclusions:**

Obesity, as well as gravidity and Hispanic race, are risk factors for lack of HPV4 vaccine adherence among young females in a safety net population.

## Introduction

There is significant evidence that obesity is a primary independent cause for lack of immune response to vaccines [1]–[6]. Obesity is highly prevalent in the US. The National Center for Health Statistics (NCHS) recently reported that **17%** of female adolescents 12–19 years old and **32%** of women 20–29 years old were obese as defined by a body mass index (BMI) of greater than 30 kg/m^2^ [7]. The overall prevalence in 12–19 year old females of morbid obesity (BMI ≥40 kg/m^2^) is 1.6% with a higher prevalence among Black youth at 3.4% [8]. When overweight (BMI: 25 to <30 kg/m^2^) females are included with the obese class, the adolescent prevalence increases to **33%** [7] and the adult 20–39 year old prevalence to **56%** [9]. The high prevalence of obesity among young females has remained consistent over the past decade [7] raising alarms that HPV4 vaccination may provide significantly less efficacy in this population of adolescent and young women.

Leptin is the biochemical and immunologic link that connects obesity to decreased vaccine immune response; it is an adipocyte-derived cytokine and a hormone [10]. Leptin levels are directly related to body fat mass, and regulate metabolic processes, reproductive and neuroendocrine functions as well as inflammation and immunity. Leptin is equally significant in female adolescents as adults [11]. Leptin influences both the innate and adaptive immune system directly, through upregulation of naïve CD4+ T cells and inhibition of CD4+ memory T cells. As obesity evolves, leptin transport becomes saturated, and immunologic processes are blocked: Th1 cell differentiation no longer occurs, and cytokine production is diminished. Lack of vaccine efficacy in obese recipients has been documented by suppressed antibody titers to seasonal flu, pandemic H1N1 flu, Hepatitis A, Hepatitis B and tetanus vaccinations [12]–[15]. In addition, hyperleptinemia is associated with more rapid declines in antibody titers over time [16].

Healthy People 2020 aims for an 80% uptake of 3 doses of HPV vaccine among adolescent females 13–15 years of age [17], as part of a public health plan to reach a reduced cervical cancer incidence of 7.1/100,000 [18] and eventually a reduced death rate to 2.2/100,000 [19]. Obese women have historically had a two-fold higher mortality from cervical cancer compared to normal weight women [20] with the death rate increasing to three fold most recently [21]. The higher death rate appears to be related to lacking of screening. Obese women are variously reported as complying with a single cervical cytology screening [22] with a substantial portion of studies reporting underuse [23]–[33], which varies by race [27], [30], [33]–[37]. More definitively, women followed over three screening periods were less likely to comply if they had a high BMI [38]. High risk HPV infections are no more likely in obese women than normal BMI women [39] and while obese women report fewer male partners in a three year period of time there was no difference in lifetime numbers of sexual partners between obese and normal weight women [40], [41].

One proven clinical value of HPV4 is its reduction in abnormal cytology screens if all three doses are received in a timely manner. The overall public health research question asks if HPV4 is ineffective in obese females; and if so, whether HPV4 vaccination in obese females is a cost effective strategy for cervical cancer prevention. The specific aim of this study was to determine if BMI influences adherence to the three dose HPV4 vaccination series in a safety net population of vulnerable and underinsured females who are already at high risk for cervical cancer. As the EMEA has approved a two dose schedule for HPV2 [42], a secondary exploratory aim is to evaluate the adherence to a two dose HPV vaccination schedule among obese women.

## Materials and Methods

This historical prospective research was approved by the Truman Medical Center (TMC) Privacy Board and by the University of Missouri Kansas City (University of Missouri-Kansas City) Adult Health Sciences Institutional Review Board as an exempt study not requiring individual verbal or written consent (#11-16e).

All females between 10–26 years of age receiving at least one dose of HPV4 between July 2006 and October 2009 were identified from electronic billing, electronic medical records (EMR) and vaccine records from the Truman Medical Center (TMC) safety net health care system. This study focused on the time frame when only HPV4 was available and advertising and general public promotions were at their highest. One nurse was assigned the HPV vaccination program and was empowered to vaccinate with standing orders making it easier for women to receive vaccination without a physician's visit. Age, race/ethnicity, pregnancy history, number of HPV4 doses received and dates of injection as well as BMI were recorded [43].

World Health Organization (WHO) definitions of BMI were used to categorize our population; underweight (<18.5 kg/m^2^), normal (between 18.5 and 24.99 kg/m^2^), overweight (between 25 and 29.99 kg/m^2^), obese class I (between 30 and 34.99 kg/m^2^), obese class II (between 35 and 39.99 kg/m^2^) and obese class III (≥40 kg/m^2^) [44].

On-time adherence to HPV4 vaccination was defined from prior publications. Early intervals were defined as less than 4 weeks between dose 1 and dose 2; less than 12 weeks between dose 2 and dose 3; or less than 24 weeks between dose 1 and dose 3. Late intervals were defined as more than 26 weeks between dose 1 and dose 2; or more than 52 weeks between dose 1 and dose 3 [45]–[49].

### Statistical Methods

Descriptive statistics included mean, standard deviation (SD), and frequencies. Inferential statistics included one way analysis of variance (ANOVA) with two-sided Tukey post hoc tests of significance between groups, as well as chi-square testing for categorical outcomes [50]. Analyses used p<0.05 as the threshold for significance, with adjustments to the p-value applied for multiple comparisons where appropriate. We derived the Cochran-Armitage test for trend by using a transformation of the linear-by-linear association provided by SPSS [51], [52]. Predictive statistics included binary logistic regression for on-time three dose HPV4 completion, reporting odds ratios (ORs), adjusted odds ratios (aORs), and 95% Confidence Intervals (CIs) [50].

## Results

Of the 1563 HPV4 recipients, 1240 (79%) had BMI information recorded. There was no difference in age, gravidity or parity between those with and without BMI recorded. Racial group proportions did differ by whether BMI information was recorded. Significantly more Hispanics (14% vs 6%, p<0.001) and significantly fewer whites (26% vs. 37%, p = 0.012) had missing BMI information.

Of the 1240 HPV4 recipients with BMI data, 4% were underweight, 33% were normal weight, 25% were overweight, 18% were obese class I, 11% were obese class II, and 9% were obese class III (38% obese overall, Table 1). The distribution of BMI categories for those adolescent females 18 years and younger is presented in the Table S1. One way ANOVA showed that there was a difference by BMI category in age, gravidity and parity (F = 7.6, 7.4, and 10.4, p<0.001 for each, respectively). Tukey post hoc testing showed that normal BMI differed from each increasing weight category for age, gravidity and parity (p≤0.04). For example, normal BMI females are significantly younger than overweight females; likewise, normal BMI females were significantly younger than obese class I females, class II and class III females. Overweight and obese women of all classes do not differ by age, gravidity or parity (p>0.05).

**Table 1:** Safety Net Study Population by Body Mass Index category.

	Underweight	Normal	Overweight	Obese Class I	Obese Class II	Obese Class III	Total
	<18.5 kg/m^2^	18.5 to <25 kg/m^2^	25 to <30 kg/m^2^	30 to <35 kg/m^2^	35 to <40 kg/m^2^	≥40 kg/m^2^	
	N = 43 (3.5%)	N = 410 (33.1%)	N = 316 (25.5%)	N = 222 (17.9%)	N = 142 (11.4%)	N = 107 (8.6%)	N = 1240 (100%)
Age^a^, yrs mean (SD)	17.5 (5.1)	20.2 (3.4)	21.2 (3.1)	21.3 (3.1)	21.2 (2.7)	21.6 (3.1)	20.8 (3.4)
Race^b^, n (%^c^)							
White	30 (6.5)	176 (38.1)	102 (22.1)	72 (15.6)	51 (11.0)	31 (6.7)	462 (37.3)
Black	10 (1.5)	201 (30.4)	183 (27.6)	126 (19.0)	76 (11.5)	66 (10.0)	662 (53.4)
Hispanic	2 (2.7)	15 (20.5)	22 (30.1)	18 (24.7)	11 (15.1)	5 (6.8)	73 (5.8)
Other	1 (2.3)	18 (41.9)	9 (20.9)	6 (14.0)	4 (9.3)	5 (11.6)	43 (3.5)
	N = 41	N = 405	N = 310	N = 215	N = 137	N = 106	N = 1214
Gravidity^a^, mean (SD)	0.6 (1.2)	1.2 (1.4)	1.5 (1.3)	1.8 (1.6)	1.7 (1.3)	1.7 (1.7)	1.5 (1.4)
Parity^a^, mean (SD)	0.4 (0.6)	0.9 (1.0)	1.2 (1.0)	1.4 (1.1)	1.3 (1.0)	1.2 (1.0)	1.1 (1.0)

a Differences in age, gravidity and parity are significant between underweight BMI females and each BMI category; and differences in age, gravidity and parity are significant between normal BMI females and all other BMI categories by one-way ANOVA.

b The proportion of white and black women significantly decreases as the BMI category increases from normal, p for trend<0.001. Hispanic women are evenly distributed among the BMI categories from normal through obese class II.

c Percentages by BMI category are per race category; percentage of total for each race is per total population.

The proportion of women of both white and black races significantly decreased as BMI increased from normal (p-for-trend <0.05). Hispanic women, on the other hand, were evenly distributed among BMI categories when obese class II and III were combined.

**Figure 1:**
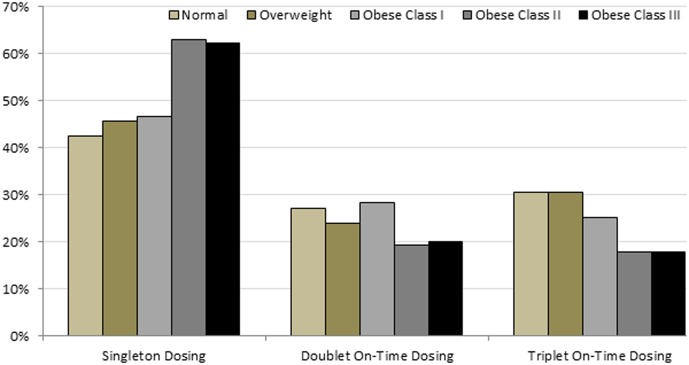
Distribution of receipt of HPV4 on time dosing by BMI categories. Women of normal and overweight BMI categories received a singleton dose of HPV4 significantly less often than women of obese class II/III (≥35 kg/m^2^) BMI categories (44% vs. 63%, p<0.001). Women of normal and overweight BMI categories received two on time doses of HPV4 significantly more often than women of obese II/III BMI categories (26% vs. 20%, p<0.05). Women of normal and overweight BMI categories received three on time doses of HPV4 significantly more often than women of obese II/III BMI categories (30% vs. 18%, p<0.001). Women of obese II/III BMI categories received a singleton HPV4 dose significantly more often than three on time doses (63% vs. 18%, p<0.001).

The number of HPV4 doses received was significantly different by BMI category (Figure 1). Women of obese class II and III BMI categories behaved in similar fashions and were grouped together for analyses. Over half of the females with BMI ≥35 kg/m^2^ (obese II/III) received only a singleton dose of HPV4. Women with a normal BMI received significantly fewer singleton HPV4 doses than obese class II/III women (42% vs. 63%, p<0.001). Conversely, women of normal BMI received significantly more on time doublet and triplet HPV4 doses than obese II/III women (27% vs 20%, p<0.043; and 30% vs 18%, p<0.001, respectively). In addition, women of obese II/III BMI categories received a singleton HPV4 dose significantly more often than three on time doses (63% vs. 18%, p<0.001).

**Figure 2:**
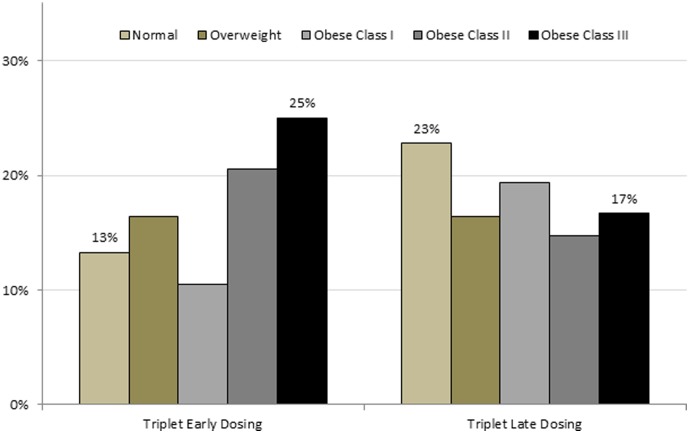
Distribution of mistimed triplet HPV4 dosing by BMI category. Among women receiving three doses of HPV4, obese III women received at least one early dose among the triplet series significantly more often than normal women (25% vs. 13%, p<0.001); and at least one late dose among the triplet series significantly less often than normal women (23% vs. 17%, p<0.001).

64% of obese II/III women received three HPV4 doses on time with 36% mistiming at least one dose. Figure 2 shows that obese II/III women received at least one early or late dose equally often (22% vs. 16%, p>0.05), whereas normal BMI women received late doses more often than early doses (23% vs. 13%, p<0.5). Among women receiving three doses of HPV4, obese III women received at least one early dose among the triplet series significantly more often than normal women (25% vs. 13%, p<0.001); and at least one late dose among the triplet series significantly less often than normal women (23% vs. 17%, p<0.001).

Overall, irrespective of BMI, nulligravid women completed three on time doses significantly more often than multigravid women: 35% (117/331) vs 18% (159/883), p<0.001. Those 18 years or younger completed three on time doses significantly more often than those older than 18: 29% (74/258) vs 21% (268/1305), p<0.01. White and black women equally completed three doses on time: 26% (143/547) and 21% (173/836), respectively, with Hispanic women completing three on time doses significantly less often 8% (9/117), p<0.01.

Univariate binary logistic regression included age, gravidity, parity, race and BMI to predict on-time triplet dosing adherence. Younger years, fewer pregnancies and births, Hispanic race and obese class II and III were independent negative predictors of on-time HPV4 triplet dosing (Table 2). Multivariate analyses included all significant univariate parameters, except for parity due to its collinear relationship with gravidity. Adjusted odds ratios showed higher gravidity and Hispanic race as significant predictors of lower chances of on-time triplet HPV4 completion.

**Table 2:** Predictors of on-time triplet dosing.

	Crude OR (95% CI)	Adjusted† OR (95% CI)
Age (10–26 years)	**0.95 (0.91, 0.99)**	0.98 (0.93, 1.02)
Gravidity	**0.72 (0.64, 0.81)**	**0.74 (0.64, 0.85)**
Parity	**0.60 (0.52, 0.71)**	**-**
Race/Ethnicity		
White	referent	referent
Black	0.77 (0.58, 1.03)	1.18 (0.85, 1.64)
Hispanic	**0.26 (0.11, 0.63)**	**0.39 (0.16, 0.95)**
BMI		
Normal: 18.5 to <25 kg/m^2^	referent	referent
Overweight: 25 to <30 kg/m^2^	1.01 (0.72, 1.41)	1.32 (0.92, 1.89)
Obese Class I: 30 to <35 kg/m^2^	0.80 (0.54, 1.18)	1.00 (0.65, 1.54)
Obese Class II: 35 to <40 kg/m^2^	**0.55 (0.33, 0.91)**	0.76 (0.45, 1.30)
Obese Class III: ≥40 kg/m^2^	**0.56 (0.32, 0.99)**	0.70 (0.38, 1.27)
Obese Class II/III combined	**0.55 (0.37, 0.83)**	-

† Adjusted for age, race, gravidity and BMI, using gravidity as a dichotomous variable n = 0 (reference) vs. n≥1.

All bolded odds ratios are significant compared to the referent category.

The relationship of BMI to gravidity in this population is interdependent. Pregnancy was common even at young ages: 74% of women had been pregnant and 70% had given birth (Table 3). For those experiencing pregnancy or birth, significantly more were categorized as obese (all classes) compared to normal BMI: 60% (380/631) vs. 40% (251/631), p<0.001; 62% (366/595) vs 38% (229/595), p<0.001, respectively. Conversely, of those never experiencing pregnancy or birth, significantly more women were of normal BMI than obese: 66% (154/232) vs 33% (78/232), p<0.001; 66% (176/268) vs 33% (92/268), p<0.001, respectively. Among obese women, significantly more had experienced pregnancy/birth than were nulligravid or nulliparous (83% vs 17%, p<0.001; 80% vs. 20%, p<0.001, respectively).

**Table 3:** BMI by Pregnancy History.

	Normal	Overweight	Obese Class I	Obese Class II	Obese Class III	All Obese	Total
	18.5 to <25 kg/m^2^	25 to <30 kg/m^2^	30 to <35 kg/m^2^	35 to <40 kg/m^2^	≥40 kg/m^2^	≥30 kg/m^2^	
	N = 405	N = 310	N = 215	N = 137	N = 106	N = 458	N = 1173
Gravidity							
n = 0	154 (38%)	70 (23%)	37 (17%)	21 (15%)	20 (19%)	**78 (17%)**	302 (26%)
n≥1	251 (62%)	240 (77%)	178 (83%)	116 (85%)	86 (81%)	**380 (83%)**	871 (74%)
Parity							
n = 0	176 (43%)	79 (25%)	41 (19%)	26 (19%)	25 (24%)	**92 (20%)**	347 (30%)
n≥1	229 (57%)	231 (75%)	174 (81%)	111 (81%)	81 (76%)	**366 (80%)**	826 (70%)

Significantly more women with one or more pregnancies/births are obese compared to nulligravid and nulliparous women, 83% vs. 17%; and 80% vs 20%, p<0.001, respectively.

**Figure 3:**
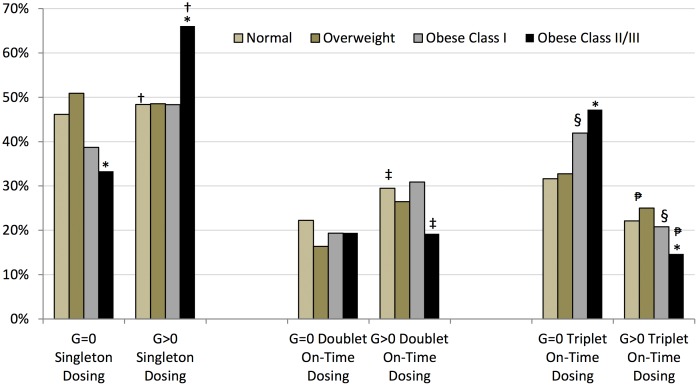
Distribution of HPV4 doses by Gravidity and BMI categories. *significant differences within obese II/III category by gravidity groups for singleton and on time triplet HPV4 dosing, 66% vs. 48% (p<0.001) for singleton dose, 15% vs. 47% (p<0.001) for on time triplet dosing. †significant difference between obese II/III vs normal BMI categories for multigravid women receiving a singleton dose, 66% vs. 48%, p<0.001. ‡significant difference between obese II/III vs normal BMI categories for multigravid women receiving on time doublet dosing, 29% vs. 19%, p<0.01. §significant difference within the obese class I category by gravidity group for on time triplet dosing, 42% vs. 21%, p<0.01. 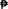
significant difference between normal/overweight vs. obese II/III BMI categories for multigravid women receiving on time triplet dosing, 24% vs 15%, p<0.01. Legend: G = 0 means the woman has never experienced pregnancy; G>0 means that the woman has experienced at least one pregnancy. On time doublet dosing means that dose 1 and 2 were received at least 4 weeks and no more six months from each other. On time triplet dosing means that on time doublet dosing occurred and there was more than 12 weeks between dose 2 and 3, more than 24 weeks and less than 52 weeks between dose 1 and 3.

Figure 3 further stratifies the distribution of HPV4 dosing by the gravidity and BMI category together. Among obese women, having experienced a pregnancy compared to being nulligravid leads to low vaccine adherence behaviors. Obese class II/III women who were multigravid received a single HPV4 dose more often than the nulligravid obese II/III women (66% vs. 48%, p<0.001). Likewise, multigravid women who were obese II/III received triplet HPV4 dosing on time less often than nulligravid women (15% vs. 47%, p<0.001). This pattern of vaccine dosing noncompliance was also true for multigravid vs nulligravid obese I women receiving three doses (21% vs. 42%, p<0.01).

Furthermore, within the multigravid class, vaccine adherence behaviors are worse for the obese than for the normal BMI woman. Significantly more multigravid obese II/III women vs normal BMI women received a singleton dose (66% vs. 48%, p<0.001). In addition, multigravid obese II/III women received on time doublet dosing significantly less often than normal BMI women (19% vs 29%, p<0.01); and, multigravid women of normal/overweight BMI completed three on time HPV4 doses more often than multigravid obese II/III women who received three on time doses (24% vs. 15%, p<0.01).

## Discussion

Our research shows that increasing BMI does worsen adherence to a three dose HPV4 vaccination series in a safety net health care population both in the number of doses received, and in the timing of doses. Moreover, our data show the nonlinear relationship of increasing BMI and gravidity on HPV4 vaccine compliance. Several studies have shown that pregnancy history and BMI influence each other in nonlinear ways, with BMI being dependent not only on parity but also on sociologic, demographic and activity indices [53]–[55]. Obesity remains an important determinant of HPV4 vaccine adherence despite lack of significance in the multivariate model. In addition, our work shows that as dosing recommendations change to a two dose series instead of three [42], obese women are still at increased risk for noncompliance compared to normal BMI women.

Several concerns about HPV4 vaccination in obese women remain. A substantial proportion of the US adolescent and young female population is obese; and the most at risk population for cervical cancer are those who seek care in safety net health care systems [56], [57]. The fact that the most at risk obese women in our safety net health care system fail to achieve full vaccination status is concerning as a single dose is insufficient to provide any protection from genital warts or cervical HPV infection.

Furthermore, the immune response among the obese to other vaccinations including HPV4 continues to be a public health issue. Early research linked the failure of antibody and long term memory response of vaccinations in the obese adolescent and adult to the needle length and angle of insertion depositing antigen in adipose tissue instead of well-vascularized muscle [58]–[60]. Recently, circulating leptin levels have been directly related to body fat mass in both female adolescents and adults [10], [11], with continued low antibody response due to leptin's interference of the innate and adaptive immune systems. If obese women mount a modified antibody response to HPV4, and then have the same rate of antibody decline documented for the general population, efficacy may be lessened. The distribution of HPV4 antibody titers in the general population varies within four years over 1000 fold for each of the four genotypes: 6, 11, 16 and 18 [61], making it possible that should a low-responder also be obese, she may not be protected as expected. In addition, the rate of complete loss of antibody detection within 5 years in 10% of the population for HPV 6 and 11 and in 35% for HPV 18 antibody titers [62] could be expected to be worse for those vaccine-compliant females who are obese.

Early dose timing also leads to a compromised immune response for many vaccines [63]. Our work shows that obese women received mistimed doses too early rather than too late, emphasizing the risk of lack of effective protection for obese women who despite receiving three doses were not appropriately primed for subsequent dosing.

Even if HPV4 vaccination is not effective for obese adolescents and women, though, cervical cancer prevention is likely just with continued screening. Obese women do not differ in genotype or incidence of HPV infection or number of lifetime sexual partners from women with normal BMI [39]–[41], and have been found to adhere to preventive screening guidelines as well as non-obese women [64]–[66] with a minority of reports suggesting the need for obese women to be encouraged to attend repeated screening exams [38]. Emphasizing the importance of cervical cancer screening at the time of HPV vaccination is mandatory for effective cervical cancer control.

In our safety net population, moving to the use of HPV2 which has proven efficacy and immunogenicity for a single dose has been a wiser use of resources than the HPV4 vaccination program; with the continued emphasis on age appropriate cervical cancer screening.

### Limitations

This work was conducted in a safety net health care system which is not generalizable to other less vulnerable populations. The proportion of young adolescents and adults already experiencing pregnancy reflects the high risk nature of the population, and may not be generalizable to the nulligravid pre-pubescent target population for HPV4 vaccination that the CDC has established. Nevertheless, it is important to identify the risk that obese multigravid women have in completing the three dose series, and continue to encourage screening participation, as they are at high risk for cervical cancer. Lastly, it is unlikely, but possible, that females usually served in our catchment area completed their HPV4 series at other health care clinics.

## Conclusions

Obese females are more likely to be noncompliant with HPV4 dosing recommendations regardless of a two or three dose regimen, and are more likely to receive dosing too early. Gravidity continues to be a powerful negative predictor of HPV4 noncompliance in both the normal and obese BMI female.

## Supporting Information

Table S1 **Safety Net Study Population by Body Mass Index category for those adolescents 18 years and younger.** BMI for the younger population studied in the safety net population.
(DOCX)
