# Supplementary material for: In a Safety Net Population HPV4 Vaccine Adherence Worsens as BMI Increases
Source: PLoS One. 2014 Jul 30;9(7):e103172. doi: 10.1371/journal.pone.0103172 (PMC4116139; doi:10.1371/journal.pone.0103172)
Supplement: Table S1 — Safety Net Study Population by Body Mass Index category for those adolescents 18 years and younger. BMI for the younger population studied in the safety net population. (DOCX) [file pone.0103172.s001.docx]

Table S1. Safety Net Study Population by Body Mass Index category for those adolescents 18 years and younger

|  | Underweight | Normal | Overweight | Obese Class I | Obese Class II | Obese Class III | Total |
| --- | --- | --- | --- | --- | --- | --- | --- |
|  | <18.5 kg/m^2^ | 18.5 to < 25 kg/m^2^ | 25 to <30 kg/m^2^ | 30 to <35 kg/m^2^ | 35 to < 40 kg/m^2^ | ≥ 40 kg/m^2^ |  |
|  | N=21 | N=90 | N=34 | N=27 | N=13 | N=12 | N=258 |
| Age, yrs mean (SD) | 12.9 (2.3) | 15.3 (1.7) | 15.1 (2.1) | 15.7 (1.4) | 15.8 (1.0) | 15.9 (1.5) | 15.2 (2.0) |
| Race, n (%^a^) |  |  |  |  |  |  |  |
| White | 16 (14.6) | 51 (46.4) | 19 (17.3) | 11 (10.0) | 7 (6.4) | 6 (5.5) | 110 (55.8) |
| Black | 3 (4.5) | 30 (44.8) | 12 (17.9) | 11 (16.4) | 5 (7.5) | 6 (9.0) | 67 (34.0) |
| Hispanic | 1 (11.1) | 3 (33.3) | 1 (11.1) | 3 (33.3) | 1 (11.1) | 0 (0) | 9 (4.6) |
| Other | 1 (9.1)) | 6 (54.5) | 2 (18.2) | 2 (18.2) | 0 (0) | 0 (0) | 11 (5.6) |
|  | N=20 | N=86 | N=34 | N=25 | N=13 | N=12 | N=243 |
| Gravidity, mean (SD) | 0.05 (0.2) | 0.33 (0.6) | 0.53 (1.0) | 0.60 (0.6) | 1.00 (0.8) | 0.75 (0.8) | 0.52 (0.7) |
| Parity, mean (SD) | 0.05 (0.2) | 0.28 (0.5) | 0.41 (0.6) | 0.52 (0.6) | 0.69 (0.6) | 0.58 (0.7) | 0.42 (0.6) |

^a^Percentages by BMI category are per race category; percentage of total for each race is per total population.
